# Complement receptor 1 polymorphisms associated with resistance to severe malaria in Kenya

**DOI:** 10.1186/1475-2875-4-54

**Published:** 2005-11-08

**Authors:** Vandana Thathy, JoAnn M Moulds, Bernard Guyah, Walter Otieno, José A Stoute

**Affiliations:** 1 The US Army Medical Research Unit and the Kenya Medical Research Institute, Nairobi, Kenya; 2 Department of Microbiology and Immunology, Drexel University College of Medicine, Philadelphia, PA, USA; 3 Department of Cellular Injury, Walter Reed Army Institute of Research, Silver Spring, MD and Department of Medicine, Uniformed Services University of Health Sciences, Bethesda, MD, USA; 4 LifeShare Blood Centers, Shreveport, LA, USA

## Abstract

**Background:**

It has been hypothesized that the African alleles *Sl2 *and *McC*^*b *^of the Swain-Langley (Sl) and McCoy (McC) blood group antigens of the complement receptor 1 (CR1) may confer a survival advantage in the setting of *Plasmodium falciparum *malaria, but this has not been demonstrated.

**Methods:**

To test this hypothesis, children in western Kenya with severe malaria-associated anaemia or cerebral malaria were matched to symptomatic uncomplicated malaria controls by age and gender. Swain-Langley and McCoy blood group alleles were determined by restriction fragment length polymorphism and conditional logistic regression was carried out.

**Results:**

No significant association was found between the African alleles and severe malaria-associated anaemia. However, children with *Sl2/2 *genotype were less likely to have cerebral malaria (OR = 0.17, 95% CI 0.04 to 0.72, P = 0.02) than children with *Sl1/1*. In particular, individuals with *Sl2/2 McC*^*a/b *^genotype were less likely to have cerebral malaria (OR = 0.18, 95% CI 0.04 to 0.77, P = 0.02) than individuals with *Sl1/1 McC*^*a/a*^.

**Conclusion:**

These results support the hypothesis that the *Sl2 *allele and, possibly, the *McC*^*b *^allele evolved in the context of malaria transmission and that in certain combinations probably confer a survival advantage on these populations.

## Background

*Plasmodium falciparum *malaria is responsible for most of the more than one million deaths that occur each year from malaria infection in Africa [1]. Most of these deaths occur as a result of complications such as severe malaria-associated anaemia (SMA) and cerebral malaria (CM) or coma [2]. The pathogenesis of these complications is poorly understood. Evidence from several studies [3-7] suggests that the complement receptor 1 (CR1, CD35) may be involved in the pathogenesis of severe malaria. However, its exact role is not known.

**Figure 1:**
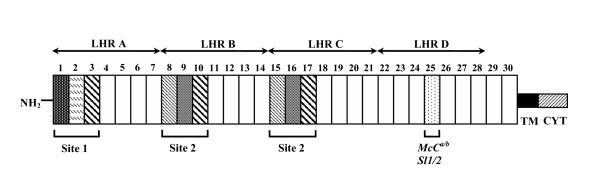
Schematic diagram of the most common structural variant of complement receptor 1 (CR1*1, F allele). The amino terminal (NH_2_) extracellular portion is composed of 30 complement control protein repeats (CCPs; vertical boxes numbered 1–30) arranged into four long homologous regions (LHRs) A-D, each composed of seven CCPs. There are two distinct functional domains each composed of three complement control protein repeats (CCPs) (vertical hatched boxes): site 1 in LHR-A (CCPs 1–3) binds mainly C4b and has convertase decay accelerating activity, and two virtually identical copies of site 2 in LHR-B (CCPs 8–10) and LHR-C (CCPs 15–17) that bind C3b and C4b, as well as PfEMP-1, and possess Factor I- cofactor activity. Functional differences in sites 1 and 2 are determined by amino acid sequence differences. Boxes with the same hatching pattern reflect near amino acid identity of the CCPs. CCP 25 (stippled box) carries the Swain-Langley (Sl) and McCoy (McC) Knops blood group antigens. The CCP repeats are followed by a transmembrane domain (TM) and a cytoplasmic tail (CYT).

CR1 is a protein ranging in *M*_*r *_from 190 to 280 kDa. It is found on erythrocytes and most leukocytes and it is divided into three to four long homologous repeat regions (LHRs) and 27 to 30 short consensus repeats (SCRs), also known as complement control protein repeats (CCPs) [8] (Figure 1). LHR-A binds C4b, whereas LHR-B and C bind both C4b and C3b. LHR-D binds mannan-binding lectin and C1q [9,10]. Together with other erythrocyte complement regulatory proteins such as decay accelerating factor (DAF, CD55) and membrane inhibitor of reactive lysis (MIRL, CD59), CR1 serves to attenuate the complement cascade. Specifically, CR1 promotes the Factor I-mediated inactivation of C3b to C3bi and promotes inactivation of C3 convertases. CR1 on erythrocytes also binds opsonized immune complexes carrying them to organs of the reticuloendothelial system for removal. Altogether, these functions serve to protect erythrocytes and other host cells from complement-mediated damage. In addition, CR1 is thought to be a receptor for the *P. falciparum *erythrocyte membrane protein 1 (PfEMP-1) [3,4]. The interaction between PfEMP-1 and CR1 may be responsible for rosetting, a phenomenon in which erythrocytes infected with *P. falciparum *late stage parasites bind to uninfected erythrocytes in vitro and has been associated with cerebral malaria in several African studies [11,12].

The CR1 gene exhibits a number of polymorphisms. There are several size variants which are felt to be the result of unequal gene crossover [13]. The most common variant has four LHRs (Figure 1). Polymorphisms that correlate with the quantitative expression of CR1 on erythrocytes of Caucasians but not of Africans have also been identified [14,15]. Lastly, the Knops blood group antigens, including the Swain-Langley (Sl), McCoy (McC) and their alleles, have been localized to the CR1 protein in LHR-D [16-18].

The study of the Sl and McC blood group antigens may offer further clues as to the role of CR1 in the pathogenesis of severe malaria. The phenotypic absence of these antigens, designated Sl -1,2 [19] and McC(a-b+) respectively, is occasionally the result of low CR1 copy numbers on the RBC or, most commonly, due to non-synonymous base substitutions [17]. The alleles responsible for the phenotypic absence are very rare in ethnic groups that are not of African descent. Thus, this observation has led to the hypothesis that the *Sl2 *and *McC*^*b *^alleles may confer a survival advantage in the face of diseases such as malaria. In support of this hypothesis Sl -1,2 erythrocytes were found to rosette less in vitro than Sl 1,-2 erythrocytes [3]. However, a recent study failed to reveal an association between *Sl2 *or *McC*^*b *^and resistance to severe malaria in The Gambia, West Africa [20]. The present study describes the findings of an investigation designed to determine whether there is an association between resistance or susceptibility to severe malaria and the Sl and McC blood group alleles in western Kenya, a region of malaria endemicity. The findings suggest that, contrary to a previous report [20], *Sl2 *does confer an advantage in the setting of malaria infection.

## Methods

### Populations and study design

Participants were recruited under human use protocols approved by the Human Subjects Research Review Board, Office of the Surgeon General, US Army, and the Ethics Review Committee of the Kenya Medical Research Institute, Nairobi, Kenya. All procedures were in accordance with the Helsinki Declaration. The study had a matched case-control design. The demographics of some of the study participants as well as the inclusion/exclusion criteria have been described before [5-7]. SMA cases, defined as children with asexual *P. falciparum *parasitaemia by Giemsa-stained thick and thin blood smear and Hb ≤ 5 g/dL, were recruited from the pediatric ward of the Nyanza Provincial General Hospital (NPGH), Kisumu, Kenya. The NPGH catchment area is the malaria holoendemic region of the Lake Victoria basin in western Kenya, where most individuals are of the Luo ethnic group. Because CM is uncommon in the Lake Victoria basin, CM cases were recruited from the pediatric ward of the Kisii District Hospital (KDH), as well as from the NPGH. KDH is located in the highlands of western Kenya where transmission is seasonal and consequently receives many more CM cases than the NPGH [21]. The predominant ethnic group in Kisii is the Abagusii. CM was defined as asexual *P. falciparum *parasitaemia by Giemsa-stained blood smear and a Blantyre coma score of ≤ 2 [22], lasting at least 30 min if there was a history of convulsions. One control with symptomatic uncomplicated malaria was matched to each case by age ± two months and gender and was identified from the outpatient clinic of the same hospital where the corresponding case was obtained. Symptomatic uncomplicated malaria was defined as a Giemsa-stained blood smear positive for asexual *P. falciparum *and an axillary temperature ≥ 37.5°C or, in the absence of the latter, two of the following signs or symptoms: nausea/vomiting, irritability, poor feeding, myalgias or headache. Children were excluded from participation if there was evidence of other concomitant serious infections (i.e. meningitis excluded by lumbar puncture, pneumonia, sepsis) or chronic illness. All children with suspected CM underwent a lumbar puncture to exclude meningitis and were enrolled if the results of Gram stain and culture were negative. In addition, because some of our studies also included the measurement of erythrocyte complement regulatory proteins, children were excluded if they had a history of blood transfusion in the three months preceding enrolment to avoid the influence of donor erythrocytes.

### Genotyping of CR1 Knops blood group polymorphisms

A whole blood sample obtained at enrolment, and prior to any blood transfusion, was used to extract DNA and/or blotted onto filter papers for later extraction. Personnel who were unaware of the group assignments of the study participants carried out DNA extraction, amplification and interpretation of restriction fragment length polymorphisms (RFLPs) patterns. DNA was extracted from whole blood using the QIAamp DNA blood extraction protocol (Qiagen, Valencia, CA). DNA extraction from filter papers was performed using saponin and Chelex as previously described [23]. Once extracted, genomic DNA was stored frozen at -20°C for later analysis. The *Sl1/Sl2 *and *McC*^*a*^/*McC*^*b *^Knops blood group alleles result from single nucleotide polymorphisms that cause specific amino acid changes[17] in exon 29 (encoding CCP 25; Figure 1). An established PCR-RFLP method was used for the detection of both SNPs [24]. Briefly, the oligonucleotide forward primer 24KnNde: 5'-ACC AGT GCC ACA CTG GAC CAG ATG GAG AAC AGC TGT TTG AGC AT-3' and reverse primer 25Rb: 5'-GGA GGA GTG TGG CAG CTT G-3', were used to amplify a 305 bp fragment of *CR1 *exon 29 by PCR. The amplification reactions were carried out on ~500 ng of genomic DNA in a 100 μl final volume containing 1× GeneAmp PCR Buffer II (Applied Biosystems, Foster City, CA), 2.5 mM MgCl_2_, 200 μM each dNTP, 0.2 μM each primer and 5 U AmpliTaq Gold^® ^DNA Polymerase (Applied Biosystems, Foster City, CA). After 9 min of initial denaturation at 95°C, the thermocycling program used consisted of 1 min denaturation at 94°C, 1 min annealing at 58°C, and 1 min extension at 72°C for 44 cycles, followed by a final extension of 10 min at 72°C. For RFLP analysis, 28 μL of each PCR product was digested with either *Mfe*I (New England Biolabs, Beverly, MA), for the detection of *Sl1*/*Sl2*, or *Bsm*I (New England Biolabs), for the detection of *McC*^*a*^/*McC*^*b*^, and the restriction fragments were resolved by agarose gel electrophoresis as previously described [24].

### Statistical analysis

All analyses were done using SPSS version 11.5 (SPSS Inc., Chicago, IL). Chi-square tests were performed to compare the observed and predicted frequencies of genotypes based on allelic frequencies in different ethnic groups. Conditional logistic regression based on matched pairs and adjusted for ethnic groups was carried out for each single locus (Sl and McC) and for the combination of the two loci. Thus, each regression model included terms both for genotype and ethnic group. All tests were two-sided with α = 0.05.

## Results

### Population demographics

Table 1 describes the total numbers of cases and controls per group and hospital. The cases and controls were well matched in age, gender and ethnic group distribution. Most of the cases and controls recruited from the NPGH were of the Luo ethnic group, whereas most of the cases and controls recruited from the KDH were of the Abagusii ethnic group.

**Table 1:** Group demographics

	Nyanza Provincial General Hospital N = 320				Kisii District Hospital N = 140	
		
	Severe Anaemia		Cerebral Malaria		Cerebral Malaria	
			
	Cases N = 137	Controls N = 137	Cases N = 23	Controls N = 23	Cases N = 70	Controls N = 70
Mean age in months (SD)	14.8(12.6)	14.5(12.2)	29.0(15.7)	30.1(15.7)	28.5(15.9)	29.0(15.8)
No. of females (%)	57(41.6)	57(41.6)	9(39.1)	9(39.1)	37(52.9)	37(52.9)
Ethnic groups (%)						
Luo	114(83.2)	115(83.9)	22(95.7)	19(82.6)	3(4.3)	4(5.7)
Abagusii	1(0.7)	0	1(4.3)	4 (17.4)	66(94.3)	65(92.9)
Luhya	15(10.9)	19(13.9)	0	0	1(1.4)	1(1.4)
Other	7(5.1)	3(2.2)	0	0	0	0

Other: Kalenjin (3), Kikuyu (3), Turkana (1), Teso (1), Nandi (1), Bukusu (1)

### Genotype frequencies

*Sl2 *and *McC*^*b *^alleles were more common in The Gambia [20], Mali [17] and western Kenya than in other non-African ethnic or racial groups where they occurred in very few numbers (Table 2). There were no significant differences between predicted and observed genotype frequencies for the two major ethnic groups, Luos and Abagusiis (data not shown), suggesting that the genotypes within these populations are in Hardy-Weinberg equilibrium.

**Table 2:** Percent of Swain-Langley and McCoy genotypes in various populations.

Populations	Swain-Langley			McCoy			Reference
			
	1/1	1/2	2/2	a/a	a/b	b/b	
Western Kenya N = 460	10	44	45	48	45	7	This paper
The Gambia N = 853	5	31	65	38	47	15	[20]
Mali N = 99	9	30	60	49	40	10	[17]
Caucasian Americans N = 100	99	1	0	100	0	0	[20]
Asian Americans N = 99	95	4	1	96	4	0	[20]
Hispanic Americans N = 100	94	6	0	95	5	0	[20]

### Sl2/2 is associated with decreased susceptibility to cerebral malaria

In order to explore whether the *Sl2 *or *McC*^*b *^alleles confer resistance or susceptibility to severe malaria compared to the *Sl1 *and *McC*^*a *^alleles, conditional logistic regression of the individual genotypes at each locus was carried out using *Sl1/1 *or *McC*^*a/a *^as reference (Table 3). There was no association between the *Sl1/2 *or *Sl2/2 *genotype and resistance or susceptibility to SMA. Although there was a trend towards decreased susceptibility to SMA for the *Sl2/2 *genotype, the association was not significant (OR = 0.65, 95% CI 0.14 to 3.06, P = 0.59). On the other hand, individuals with the *Sl2/2 *genotype were less likely to have cerebral malaria than individuals with *Sl1/1 *(OR = 0.17, 95% CI 0.04 to 0.72, P = 0.02). This finding was independent of the hospital site since inclusion of this variable in the model did not affect the results. There was no significant effect of the *McC*^*a/b *^or *McC*^*b/b *^genotypes, when averaged over the different *Sl *alleles, on resistance or susceptibility to CM.

**Table 3:** Conditional logistic regression based on matching to compare individual genotypes.

	Severe Anaemia					Cerebral Malaria				
		
Genotype	No. of cases (%)	No. of controls (%)	OR	95% CI	*P*	No. of cases (%)	No. of controls (%)	OR	95% CI	*P*
Sl1/1	11(8)	10(7)	Ref	-	-	17(18)	10(11)	Ref	-	-
Sl1/2	63(46)	57(42)	1.08	0.24 to 4.93	0.92	44(47)	40(43)	0.49	0.12 to 2.05	0.32
Sl2/2	63(46)	70(51)	0.65	0.14 to 3.06	0.59	32(34)	43(46)	0.17	0.04 to 0.72	0.02
McC^a/a^	61(45)	61(45)	Ref	-	-	51(55)	49(53)	Ref	-	-
McC^a/b^	65(47)	66(48)	0.93	0.41 to 2.11	0.86	37(40)	40(43)	1.39	0.55 to 3.52	0.49
McC^b/b^	11(8)	10(7)	1.38	0.29 to 6.57	0.68	5(5)	4(4)	3.52	0.43 to 29.06	0.24

OR = Odds Ratio, CI = Confidence interval, Ref = Reference Category

### *Sl2/2 McC*^*a/b *^genotype is associated with decreased susceptibility to cerebral malaria

In order to determine the effect of the different genotype combinations at the Swain-Langley and McCoy loci on the susceptibility to severe malaria conditional logistic regression was carried out using the *Sl1/1 McC*^*a/a *^genotype as reference (Table 4). Only six of the ten possible *Sl/McC *genotype combinations were found. Although it is not known whether the haplotypes for the group with genotype *Sl1/2 McC*^*a/b *^are 1a/2b or 1b/2a, it was assumed that these individuals were 1a/2b because genotypes *Sl1/1 McC*^*a/b*^, *Sl1/1 McC*^*b/b *^and *Sl1/2 McC*^*b/b *^were not found in the population, suggesting that haplotype 1b is extremely rare or does not exist. Table 4 shows that among CM cases and controls with *McC*^*a/a *^background the susceptibility to CM (OR) decreased from *Sl1/2 *to *Sl2/2*, suggesting a gene dose effect. The strongest association was seen with genotype *Sl2/2 McC*^*a/b *^(OR = 0.18, 95% CI = 0.04 to 0.77, P = 0.02). Interestingly, heterozygosity at both loci or homozygozity for *Sl2 *and *McC*^*b *^did not show any significant benefit.

**Table 4:** Conditional logistic regression to compare genotype combinations to the non-African genotype *Sl1/1 McC*^*a/a*^.

	Severe Anaemia					Cerebral Malaria				
		
Genotype	No. of cases (%)	No. of controls (%)	OR	95% CI	*P*	No. of cases (%)	No. of controls (%)	OR	95% CI	*P*
Sl1/1 McC^a/a^	11(8)	10(7)	Ref	-	-	17(18)	10(11)	Ref	-	-
Sl1/2 McC^a/a^	33(24)	34(25)	0.93	0.20 to 4.37	0.93	23(25)	25(27)	0.40	0.09 to 1.77	0.23
Sl2/2 McC^a/a^	17(12)	17(12)	0.86	0.17 to 4.43	0.86	11(12)	14(15)	0.24	0.05 to 1.19	0.08
Sl2/2 McC^a/b^	35(26)	43(31)	0.53	0.12 to 2.34	0.40	16(17)	25(27)	0.18	0.04 to 0.77	0.02
Sl1/2 McC^a/b^	30(22)	23(17)	1.19	0.26 to 5.40	0.82	21(23)	15(16)	0.93	0.19 to 4.60	0.93
Sl2/2 McC^b/b^	11(8)	10(7)	0.87	0.15 to 5.14	0.88	5(5)	4(4)	0.65	0.07 to 6.07	0.71

OR = Odds Ratio, CI = Confidence Interval, Ref = Reference category

## Discussion

The present study has shown that individuals with the *Sl2/2 *genotype that confers the Sl -1,2 phenotype have decreased susceptibility to CM compared to individuals with the *Sl1/1 *genotype. This finding was independent of ethnicity, of the genotype at the *McC *locus and of the hospital site where the cases and controls were recruited. Among the combined genotypes, a trend of increasing benefit was observed in the background of *McC*^*a/a *^from *Sl1/2 *to *Sl2/2*, which is consistent with a gene dose effect. Although the level of statistical significance may be influenced by a larger sample size in the *Sl2/2 *category, the association nevertheless appears strongest with the acquisition of one *McC*^*b *^allele (Table 4).

The finding that genotype *Sl2/2 McC*^*a/b *^might be the one that most influences the level of susceptibility to CM may seem counterintuitive since in Table 3* McC*^*a/b *^did not show any significant advantage and perhaps showed a trend towards a detrimental effect relative to *McC*^*a/a*^. However, in the analysis in Table 3 the reference genotype *McC*^*a/a *^contains three variants: *Sl1/1 McC*^*a/a*^, *Sl1/2 McC*^*a/a*^, and *Sl2/2 McC*^*a/a *^with the latter two both showing indications of decreased susceptibility due to the presence of *Sl2 *(Table 4). The lack of significant benefit from *Sl2/2 *in SMA probably reflects the distinct pathogenic mechanisms between this condition and CM, but this does not exclude a role for CR1 in the pathogenesis of SMA.

In contrast to this study, a previous case-control study in The Gambia [20] failed to demonstrate any significant association between *Sl2/2 *and resistance to severe malaria. The divergence in findings between the present and the previous study could be explained by important methodological differences. In the Gambian study controls did not have malaria. This may have led to an overrepresentation of non-protective genotypes in the controls because these children did not have the most important risk factor for severe malaria, i.e. malaria. Further, cases and controls were not matched by age in the Gambian study, and in fact, cases were older than controls by an average of almost one year. In addition, it is possible that differences in the pathogenesis of severe malaria between the two sites on the basis of other genetic factors and/or differences in the malaria transmission patterns may exist.

The mechanism by which the *Sl2 *and *McC*^*b *^alleles could confer protection from severe malaria is not known, since these polymorphisms are located near the C-terminus of the CR1 molecule in an area that is outside the binding sites for C3b, C4b, and PfEMP-1. Nonetheless, there are a number of potential explanations based on the knowledge of CR1 function and the nature of the amino acid substitutions that take place. The *Sl2 *allele is the result of the substitution of glycine, a neutral amino acid, for the basic amino acid arginine at position 1601 (R1601G), whereas the *McC*^*b *^allele is the result of the substitution of the acidic amino acid glutamic acid for the basic amino acid lysine at position 1590 (K1590E) [17]. These modifications may inflict significant conformational or charge changes to the rest of the molecule and may consequently impact on the conformation and function of the C3b and C4b binding sites and/or the PfEMP-1 binding site. Accordingly, Sl -1,2 erythrocytes have been reported to rosette less than Sl 1,-2 erythrocytes [3]. In addition, it is known that CR1 molecules are aggregated on the erythrocyte surface and this aggregation is felt to be critical to the binding affinity of CR1 for C3b and C4b [25]. Therefore, it is possible that the amino acid substitutions not only induce conformational changes to the individual CR1 molecules but also affect the ability of functional aggregates to form due to conformational incompatibility or charge interference between different CR1 molecules. This could explain why some combinations of these alleles, such as heterozygosity at both loci or homozygosity for both *Sl2 *and *McC*^*b*^, did not offer any significant advantage.

Although the current expectation is that CR1 may influence the development of severe malaria by virtue of its function as a regulator of the complement cascade on RBCs or by its direct interaction with the infected RBCs leading to rosette formation, CR1 may exert its effect on malaria in other ways that are not yet understood. In addition to being present on RBCs, CR1 is also present on follicular dendritic cells, macrophages and T and B lymphocytes and, therefore, may also have immunoregulatory functions that affect the development of immunity against malaria [13]. Mice deficient in CR1/CR2 have depressed humoral immune responses [26]. Further studies will be required to explore this possibility.

Further evidence that the role of CR1 in the pathogenesis of severe malaria may not be as expected is found in a recent case-control study in Papua New Guinea that aimed to determine whether there was an association between individuals who had the low CR1 expression allele (L) and resistance to severe malaria [27]. Contrary to expectation, the association was found only in heterozygotes (HL), and not in homozygotes (LL), which presumably had the lowest CR1 levels. In addition, although there was an association with resistance to severe malaria as a whole, there was no apparent association between these polymorphisms and CM. These results suggest that the relationship between the number of CR1 molecules on the RBC surface and severe malaria is not a straightforward one.

The results obtained in the study presented here have biological significance and are further supportive evidence of the importance of CR1 in the pathogenesis of severe malaria. Whatever the mechanism is by which CR1 is involved in the pathogenesis of malaria, knowledge of which alleles are associated with resistance or susceptibility to severe malaria will make it possible to observe the relationship between structure and function and susceptibility to severe malaria.

## Conclusion

The results support the conclusion that the *Sl2 *allele and, possibly, the *McC*^*b *^allele have evolved in the context of malaria transmission in Africa and that in certain combinations probably confer a survival advantage to malaria endemic populations.

## Authors' contributions

Vandana Thathy, assisted by Bernard Guyah, optimized and carried out the PCR, performed the restriction enzyme digestions and interpreted the RFLP results. JoAnn M. Moulds developed the PCR-RFLP assay. Walter Otieno supervised the clinical evaluations of the study participants. José A. Stoute was the principal investigator who designed the study, wrote the protocol and drafted the original manuscript that was reviewed and edited by all authors.

## References

[B1] Organization WH (2002). World Health Report.

[B2] (2000). Severe falciparum malaria. World Health Organization, Communicable Diseases Cluster. Trans R Soc Trop Med Hyg.

[B3] Rowe JA, Moulds JM, Newbold CI, Miller LH (1997). P. falciparum rosetting mediated by a parasite-variant erythrocyte membrane protein and complement-receptor 1. Nature.

[B4] Rowe JA, Rogerson SJ, Raza A, Moulds JM, Kazatchkine MD, Marsh K, Newbold CI, Atkinson JP, Miller LH (2000). Mapping of the region of complement receptor (CR) 1 required for Plasmodium falciparum rosetting and demonstration of the importance of CR1 in rosetting in field isolates. J Immunol.

[B5] Waitumbi JN, Opollo MO, Muga RO, Misore AO, Stoute JA (2000). Red cell surface changes and erythrophagocytosis in children with severe Plasmodium falciparum anemia. Blood.

[B6] Stoute JA, Odindo AO, Owuor BO, Mibei EK, Opollo MO, Waitumbi JN (2003). Loss of red blood cell complement regulatory proteins and increased levels of circulating immune complexes are associated with severe malarial anemia. J Infect Dis.

[B7] Waitumbi JN, Donvito B, Kisserli A, Cohen JH, Stoute JA (2004). Age-related changes in red blood cell complement regulatory proteins and the susceptibility to severe malaria. J Infect Dis.

[B8] Krych-Goldberg M, Atkinson JP (2001). Structure-function relationships of complement receptor type 1. Immunol Rev.

[B9] Ghiran I, Barbashov SF, Klickstein LB, Tas SW, Jensenius JC, Nicholson-Weller A (2000). Complement receptor 1/CD35 is a receptor for mannan-binding lectin. J Exp Med.

[B10] Klickstein LB, Barbashov SF, Liu T, Jack RM, Nicholson-Weller A (1997). Complement receptor type 1 (CR1, CD35) is a receptor for C1q. Immunity.

[B11] Carlson J, Helmby H, Hill AV, Brewster D, Greenwood BM, Wahlgren M (1990). Human cerebral malaria: association with erythrocyte rosetting and lack of anti-rosetting antibodies. Lancet.

[B12] Rowe A, Obeiro J, Newbold CI, Marsh K (1995). Plasmodium falciparum rosetting is associated with malaria severity in Kenya. Infect Immun.

[B13] Ahearn JM, Fearon DT (1989). Structure and function of the complement receptors, CR1 (CD35) and CR2 (CD21).. Adv Immunol.

[B14] Herrera AH, Xiang L, Martin SG, Lewis J, Wilson JG (1998). Analysis of complement receptor type 1 (CR1) expression on erythrocytes and of CR1 allelic markers in Caucasian and African American populations. Clin Immunol Immunopathol.

[B15] Rowe JA, Raza A, Diallo DA, Baby M, Poudiougo B, Coulibaly D, Cockburn IA, Middleton J, Lyke KE, Plowe CV, Doumbo OK, Moulds JM (2002). Erythrocyte CR1 expression level does not correlate with a HindIII restriction fragment length polymorphism in Africans; implications for studies on malaria susceptibility. Genes Immun.

[B16] Moulds JM, Nickells MW, Moulds JJ, Brown MC, Atkinson JP (1991). The C3b/C4b receptor is recognized by the Knops, McCoy, Swain-langley, and York blood group antisera. J Exp Med.

[B17] Moulds JM, Zimmerman PA, Doumbo OK, Kassambara L, Sagara I, Diallo DA, Atkinson JP, Krych-Goldberg M, Hauhart RE, Hourcade DE, McNamara DT, Birmingham DJ, Rowe JA, Moulds JJ, Miller LH (2001). Molecular identification of Knops blood group polymorphisms found in long homologous region D of complement receptor 1. Blood.

[B18] Rao N, Ferguson DJ, Lee SF, Telen MJ (1991). Identification of human erythrocyte blood group antigens on the C3b/C4b receptor. J Immunol.

[B19] Daniels GL, Cartron JP, Fletcher A, Garratty G, Henry S, Jorgensen J, Judd WJ, Levene C, Lin M, Lomas-Francis C, Moulds JJ, Moulds JM, Moulds M, Overbeeke M, Reid ME, Rouger P, Scott M, Sistonen P, Smart E, Tani Y, Wendel S, Zelinski T (2003). International Society of Blood Transfusion Committee on terminology for red cell surface antigens: Vancouver Report. Vox Sang.

[B20] Zimmerman PA, Fitness J, Moulds JM, McNamara DT, Kasehagen LJ, Rowe JA, Hill AV (2003). CR1 Knops blood group alleles are not associated with severe malaria in the Gambia. Genes Immun.

[B21] Marsh K, Snow RW (1999). Malaria transmission and morbidity. Parassitologia.

[B22] Molyneux ME, Taylor TE, Wirima JJ, Borgstein A (1989). Clinical features and prognostic indicators in paediatric cerebral malaria: a study of 131 comatose Malawian children. Q J Med.

[B23] Farnert A, Arez AP, Correia AT, Bjorkman A, Snounou G, do Rosario V (1999). Sampling and storage of blood and the detection of malaria parasites by polymerase chain reaction. Trans R Soc Trop Med Hyg.

[B24] Moulds JM, Thomas BJ, Doumbo O, Diallo DA, Lyke KE, Plowe CV, Rowe JA, Birmingham DJ (2004). Identification of the Kna/Knb polymorphism and a method for Knops genotyping. Transfusion.

[B25] Paccaud JP, Carpentier JL, Schifferli JA (1990). Difference in the clustering of complement receptor type 1 (CR1) on polymorphonuclear leukocytes and erythrocytes: effect on immune adherence. Eur J Immunol.

[B26] Molina H, Holers VM, Li B, Fung Y, Mariathasan S, Goellner J, Strauss-Schoenberger J, Karr RW, Chaplin DD (1996). Markedly impaired humoral immune response in mice deficient in complement receptors 1 and 2. Proc Natl Acad Sci U S A.

[B27] Cockburn IA, Mackinnon MJ, O'Donnell A, Allen SJ, Moulds JM, Baisor M, Bockarie M, Reeder JC, Rowe JA (2004). A human complement receptor 1 polymorphism that reduces Plasmodium falciparum rosetting confers protection against severe malaria. Proc Natl Acad Sci U S A.

